# Crosstalk between Acupuncture and NF-*κ*B in Inflammatory Diseases

**DOI:** 10.1155/2020/7924985

**Published:** 2020-06-08

**Authors:** Dan Luo, Li Liu, Qi Huang, Hai-ming Zhang, Zhao-min Yu, Man Hu, Jin-xiao Li, Feng-xia Liang, Rui Chen

**Affiliations:** ^1^Department of Respiratory, Wuhan No. 1 Hospital, Wuhan 430022, China; ^2^Department of Pathology, Wuhan No. 1 Hospital, Wuhan 430022, China; ^3^Department of Rehabilitation, The Central Hospital of Wuhan, Tongji Medical College, Huazhong University of Science and Technology, Wuhan 430014, China; ^4^Department of Oncology, Integrated Traditional Chinese and Western Medicine, The Central Hospital of Wuhan, Tongji Medical College, Huazhong University of Science and Technology, Wuhan 430014, China; ^5^Department of Oncology, Hubei Province Hospital of Integrated Traditional Chinese and Western Medicine, Wuhan 430015, China; ^6^Department of Integrated Traditional Chinese and Western Medicine, Union Hospital, Tongji Medical College, Huazhong University of Science and Technology, Wuhan 430022, China; ^7^Department of Acupuncture and Moxibustion, Hubei University of Traditional Chinese Medicine, Wuhan 430061, China

## Abstract

Acupuncture has been used in China for thousands of years and concerned as a typical alternative medicine in inflammatory diseases nowadays. The nuclear factor-*κ*B (NF-*κ*B) transcription factor is an important regulator of inflammation. In this article, we discuss the role of acupuncture in NF-*κ*B pathways and also present the acupoints selection, acupuncture administration, and related inflammation diseases and models from previous studies to bring readers close to a more complete understanding of the mechanisms between acupuncture and NF-*κ*B in inflammatory diseases.

## 1. Introduction

Acupuncture is an oldest part of traditional Chinese medicine (TCM) with a history of more than three thousand years [1]. In recent decades, the evidence supported acupuncture which could be used as an efficient alternative therapy [2]. In the TCM theory, the combination of specific acupoints can relieve inflammatory symptoms, and it is widely studied in pain [3], obesity [4], and digestive system [5] diseases.

In mammals, the nuclear factor-*κ*B (NF-*κ*B) represents a family of inducible transcription factors [6]. Most of the inflammatory responses caused by infection, inflammatory cytokines, and engagement of antigen receptors are associated with the activation of NF-*κ*B [7].

The canonical pathway of NF-*κ*B activation consists of a series of steps [6]. First, TGF*β*-activated kinase 1 could be activated in the intracellular signaling cascade and further activates a trimeric I*κ*B kinase (IKK), which is composed of IKK*α*, *β*, and *γ*. In turn, the IKK complex will degrade I*κ*B family members with ubiquitination and phosphorylation, resulting in NF-*κ*B release and nuclear translocation. In general, the degradation of I*κ*B, a key inhibition of NF-*κ*B, is crucial for NF-*κ*B activation and acts as a potential anti-inflammatory target [8].

MicroRNAs (miRNAs) are a kind of small endogenous RNAs, which could silence genes posttranscriptionally [9]. The communication of miRNAs and NF-*κ*B was identified during inflammation [10]. In ulcerative colitis (UC), the contribution of miRNA in lowering IKK*α* expression levels could result in the activation of the NF-*κ*B pathway [11].

Other classes of molecules could also act as a regulator of NF-*κ*B. In inflammation and metabolic disorders, SIRT1 shows an antagonistic role of NF-*κ*B [12]. P38, usually activated in proliferating cells, could act as an upstream agent of the NF-*κ*B pathway in inflammatory response [13].

Not only in inflammation, but NF-*κ*B could also contribute in many other cellular processes, including proliferation, apoptosis, and immune response [14], that may be a core of cellular homeostasis [15]. The TCM theory believes that harmony is the most important goal of health. Hence, we reviewed current literatures, trying to clarify the detail role of acupuncture and NF-*κ*B in inflammatory diseases.

## 2. Methods

The research questions were combined into three key focus areas. (1) The mechanisms on how acupuncture could modulate NF-*κ*B. (2) The detail of acupuncture regulating NF-*κ*B in inflammation response. (3) The evidences that acupuncture could cure inflammatory diseases through NF-*κ*B pathways.

Systematic searches were performed in the PubMed database with key words related to “acupuncture,” “electroacupuncture,” “NF-*κ*B,” “I*κ*B,” and “P65”. Papers published before 13 April 2020, present in English, containing clear intervention by acupuncture and providing objective outcome using randomized controlled trials were selected.

## 3. Results

This review contains 8 articles which reveal the mechanisms on how acupuncture could regulate NF-*κ*B, 9 articles which introduce the detail of acupuncture regulating NF-*κ*B in inflammation response, and 22 articles which show evidence that acupuncture could ameliorate inflammatory diseases through NF-*κ*B pathways. All of them are animal studies.

### 3.1. Mechanisms of Acupuncture Regulating NF-*κ*B

**Figure 1:**
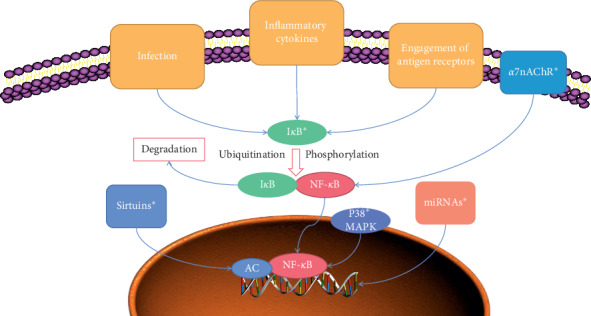
Mechanisms of acupuncture regulating NF-*κ*B. ^*∗*^Acupuncture could inhibit NF-*κ*B through sirtuins, P38, *α*7nAChR, miRNAs, and I*κ*B in this review. Infection, inflammatory cytokines, and engagement of antigen receptors can activate NF-*κ*B leading to inflammation responses. I*κ*B is a key to inhibit this progress. Current evidence showed that acupuncture could modulate IKK*α* and sirtuins to decrease the activation of NF-*κ*B. The role of miRNAs in inflammation is multiple, and acupuncture could dual-regulate the level of different miRNAs based on their function. As a result, acupuncture could be concerned as an efficient NF-*κ*B antagonist, and there may be other complex mechanisms which are still unclear.

All articles in this review promote that acupuncture could inhibit the activation of NF-*κ*B, but only a few of them investigated the detailed mechanisms. Based on current studies, acupuncture may modulate NF-*κ*B through different ways (Figure 1).

In the upstream of NF-*κ*B, *α*7nAChR [16], Sirt1 [17], and P38 [18] were identified as targets of acupuncture in NF-*κ*B pathways. Meanwhile, miRNAs also play a great role in this process. The regulation of acupuncture on miRNAs may be different depending on their function. Studies observed that acupuncture decreases miR-155 and miR-21 and increase miR-146a, which leads to inhibition of NF-*κ*B [19], and the decrease of miR-155 by acupuncture is also observed [20].

When it comes to the canonical pathway of NF-*κ*B activation, acupuncture shows great potential in inhibiting the degradation of I*κ*B [21]. Wei et al. observed that acupuncture enhances the expression of IKK*α* against the activation of NF-*κ*B [22].

### 3.2. Further Intervention of Acupuncture on Inflammation Responses after Regulating NF-*κ*B

**Figure 2:**
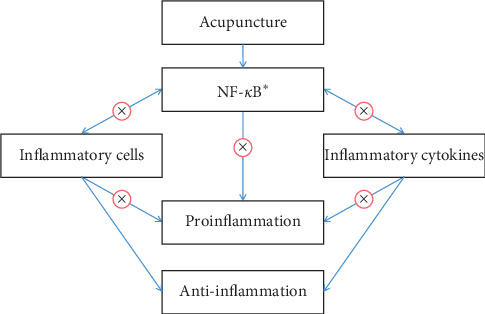
Further intervention of acupuncture on inflammation responses after regulating NF-*κ*B. ^*∗*^Acupuncture could inhibit the activation of NF-*κ*B and further regulates inflammatory cells and inflammatory cytokines to relieve inflammatory response in this review. Inflammation cells include Th17 and Treg cells, macrophages 1 and macrophages 2 cells, and mast cells. Inflammatory cytokines include IL-1, IL-1*β*, IL-6, IL-10, IL-12, IL-13, TNF-*α*, and MCP-1. These inflammation factors have pro- or anti-inflammation function. After downregulation of NF-*κ*B by acupuncture, these inflammatory factors present a tendency of anti-inflammation.

In most studies, NF-*κ*B has been used as a marker to measure the efficacy of acupuncture in inflammation. Meanwhile, some research studies suggest that the regulation of NF-*κ*B by acupuncture could induce the further intervention of inflammatory cells or inflammatory cytokines to relieve inflammatory response (Figure 2).

Wei et al. investigated that acupuncture could modulate the activity of Th17 and Treg cells and the number of CD4 + IL-17A + cells and CD4 + Foxp3 + cells in asthma rats [22]. Wang et al. identified that the anti-inflammation effect of acupuncture on allergic diseases is targeting inflammatory cytokines including IL-6, TNF-*α*, IL-13, and MCP-1 in mast cells [20]. In a study performed by Wu et al., acupuncture attenuates IL-1*β*, IL-6, TNF-*α*, and MMP-3 on knee osteoarthritis [23]. In research studies about obesity, acupuncture could regulate inflammatory cytokines, such as TNF-*α*, IL-6, and IL-1*β* [24] and inflammatory cells, such as macrophages [17]. Similar phenomenon was observed in acupuncture treating pruritus [25]. Liu et al. [26] and Xue et al. [27] also found that acupuncture could act against the release of proinflammatory cytokines by the regulation of NF-*κ*B.

### 3.3. Inflammatory Diseases Could Be Ameliorated by Acupuncture through NF-*κ*B Pathways

The current evidence supports that acupuncture could be administrated as an obvious NF-*κ*B inhibitor to treat extensive inflammatory diseases (Table 1).

The strategy of acupoints selection in different diseases was basically according to the TCM theory. But, some specific acupoints with high frequency were used. For example, due to its enhanced immune function in the TCM theory, ST36 was widely applied in different studies with the same effect on inhibiting NF-*κ*B and relieving inflammation.

In acupuncture delivery, manual acupuncture (MA) or electroacupuncture (EA) was adopted in different studies. The overall data indicate that both MA and EA could decrease NF-*κ*B and suppress inflammation. There is not enough evidence to show a significant difference in regulating NF-*κ*B between MA and EA, including different waveform, frequency, and current.

Analyzing all the results as a whole, acupuncture has an optimistic effect on the inflammatory response in various organs, tissues, and cells. Whether it is a 'long-term or short-term treatment, all subjects are benefited from acupuncture treatment. Since all the studies were conducted on animals, the side effects of acupuncture are unclear.

## 4. Discussion

Nowadays, the curative effect of acupuncture is recognized all around the world. There are plenty of clinical trials, which demonstrate acupuncture as a useful alternative medical therapy in inflammatory diseases [39] or inflammatory responses in diseases [40].

NF-*κ*B has been studied in inflammation for a long time, and it is found closely associated with multiple signaling pathways [8]. NF-*κ*B could regulate a variety of cellular mechanisms, making it a key factor in inflammation, immunity, and even tumors [41].

In this article, we discussed current studies about acupuncture, which regulate NF-*κ*B pathways in inflammatory diseases. The evidence supplies that acupuncture effectively inhibits the activation of NF-*κ*B. However, compared with the complexity of NF-*κ*B pathways, the mechanism of acupuncture in inhibiting NF-*κ*B in current studies is still superficial. The regulation of acupuncture on NF-*κ*B was only identified on miRNAs, sirtuins, and other upstream agents and I*κ*B in the canonical pathway. This information may explain how acupuncture could modulate NF-*κ*B pathways. But considering future research, there are much more unknown mechanisms, such as the noncanonical pathway, subunit target of NF-*κ*B, and other combined signal pathways. In addition, the subsequent modification on inflammatory cytokines and inflammatory cells may be crucial to explain the detail of acupuncture and NF-*κ*B on inflammation. Some cell experiments, which investigated the complex mechanisms of acupuncture regulating NF-*κ*B pathways, provide us a deeper understanding [42]. But there was no acupuncture intervention in their protocol, making them unsuitable to this review.

The intervention of acupuncture on NF-*κ*B pathways is effective on treating various inflammatory diseases in animal models. We also searched clinical trials with this topic. Although present trials did not involve the NF-*κ*B pathway, the efficacy of acupuncture in some inflammatory diseases, including asthma [43], chronic atrophic gastritis [44], cognitive impairment [45], chronic obstructive pulmonary disease [46], and brain damage [47], was recognized.

One of the objectives of this article is to better apply acupuncture in the clinical practice of inflammatory diseases. The selection of acupoints is crucial for the acupuncture therapy in TCM. There may be some broad-spectrum anti-inflammation acupoints such as ST-36, which could be used in various inflammatory diseases. To our knowledge, different waveform, frequency, and current of EA may have different therapeutic effects. We have detailed the setting of EA delivery, but it is hard to recommend an optimal option of EA for treating inflammation based on the current findings.

There are some limitations that need to be considered. First, in order to ensure the authenticity and reliability of the conclusions, only a small number of studies were identified based on our searching criteria. Although the inhibitory effect of acupuncture on NF-*κ*B was significant, there were insufficient duplication experiments to explain the detail of this process. Many unknown mechanisms need to be investigated. Second, publication bias may also exist. Last, all studies in this review were based on animals. When it comes to human, higher levels of evidence and treatment protocols are necessary. And because of the diversity and particularity of inflammatory diseases, all possible adverse reactions in acupuncture treatment should be noticed.

## 5. Conclusion

This article provided an overview of the crosstalk between acupuncture and NF-*κ*B in inflammatory diseases. A variety of studies with different diseases and models support acupuncture as an efficient NF-*κ*B antagonist and modulate inflammatory cytokines and inflammatory cells to relieve inflammation. It may help us in understanding the role of acupuncture and NF-*κ*B in inflammatory diseases better. More studies are still needed in the future to provide high-level evidence.

## Figures and Tables

**Table 1:** Inflammatory diseases could be ameliorated by acupuncture through NF-*κ*B pathways.

Disease	Models	Acupoints	Acupuncture administration	Main results	Reference
Asthma	OVA-induced mouse asthma model	GV14, BL12, BL13	MA, 30 mins each day, every other day for 4 weeks	Acupuncture attenuated inflammation and inhibited Th17 and the Treg activity	[22]
Allergic contact dermatitis	DNCB-induced mouse atopic dermatitis	LI11	MA, 8 days	Acupuncture treatment is effective in alleviating allergic contact dermatitis by reducing proinflammatory cytokines and proteins	[28]
	DNFB-induced mouse atopic dermatitis	ST36	EA, continuous waves, 2 Hz and 1 mA for 5 min, 2 Hz and 1.5 mA for 5 min, and 2 Hz and 2 mA for 20 min each day, 7 days	EA treatment inhibits NF-*κ*B and AP-1 activation, as well as promotes the negative feedback regulation of IL-33 signaling via targeting miR-155 in mast cells	[20]
CAG	MNNG-induced CAG rat model	ST36, CV12	MA, 15 mins each day, 60 days	Acupuncture downregulate NF-*κ*B p65, miR-155, and miR-21 and upregulate miR-146a expression in CAG rats	[19]
Cognitive impairment	Cerebral I/R-injured rat model	DU20, DU24	EA, disperse waves, 1 and 20 Hz, 30 mins each day, 10 days	Electroacupuncture ameliorates cognitive impairment through inhibition of NF-*κ*B-mediated neuronal cell apoptosis	[29]
COPD	Smoking-induced COPD rat model	ST36, BL13	EA, alternating waves, 10/50 Hz and 2 mA for 30 mins each day, 7 days	EA treatment can reduce the lung inflammatory response and improve the lung function in COPD	[16]
Depression	Chronic unpredictable stress rat model of depression	GV20, PC6	MA, 10 mins each day, every other day for 4 weeks	Acupuncture markedly inhibited the activation of NF-*κ*B in the brain regions	[30]
		GV20, GV29	MA, 20 mins each day, 28 days	The antidepressant effect of acupuncture is effective and has a multitarget characteristic, which may be related to amino acid metabolism and inflammatory pathways	[31]
HIBD	HIBD rat model	DU14, DU20	EA, 2–100 Hz and 3 mA for 30 mins each day, 14 days	EA against hypoxic-ischemic brain damage in rats via NF-*κ*B/neuronal nitric oxide synthase	[32]
Neuropathic pain	PTX-induced neuropathic pain rat model	ST36	EA, continuous waves, 10 Hz and 1 mA for 10 mins each day, every other day for 15 days	EA treatment attenuates PTX-induced neuropathic pain via inhibiting spinal glia and the TLR4/NF-*κ*B pathway	[33]
Neurodegeneration disease	Telomerase-deficient mice	ST36	MA or EA, 7 days	EA could specifically ameliorate the spatial learning and memory capability for telomerase-deficient mice through the activation of TrkB and NF-*κ*B than MA	[34]
Obesity	Leptin deficient mice	ST36	EA, continuous waves, 2 Hz, 0.5 and 1 mA for 10 mins each day, three times weekly for one or two consecutive weeks	EA prevents weight gain through modulation of HIF-1*α*-dependent pathways and inflammatory response in obese adipose tissues	[17]
	High fat diet-induced obesity rat model	ST36, ST40, CV3, CV4	EA, continuous waves, 2 Hz and 1 mA for 10 mins each day, three times weekly for 8 weeks	EA prevents inflammation through activation of Sirt1	[24]
OA	Surgery-induced OA rabbit model	ST35, EX-LE5	EA, square waves, 2 Hz and 100 Hz alternating polarity for 30 mins each day, 8 weeks	EA treatment may delay cartilage degeneration by downregulating inflammatory factors through the NF-*κ*B signaling pathway	[23]
Pruritus	Morphine-induced pruritus mouse model	LI11, SP10	EA, square waves, 2/15 Hz and 2 mA for 30 mins each day, 5 days	EA preconditioning improved pruritus through the TLR2/4-MyD88-NF-*κ*B pathway	[25]
RA	Surgery-induced RA rabbit mode	ST35, EX-LE5	EA, continuous waves, 2 Hz and 2 mA for 30 mins each day, 4 weeks	EA can reduce the expression of TLR4, MYD88, and NF-*κ*B, which play an important role in treatment of adjuvant arthritis	[35]
Stroke	MACo rat model	GV20, GV14	EA, amplitude-modulated waves, 5 Hz and 2.7–3.0 mA for 25 mins each day, 6 days	EA subacute phase cerebral I/R injuries by reducing S100B-mediated neurotoxicity	[18]
		LI11, ST36	EA, dilatational waves, 1–20 Hz and 2.7–3.0 mA for 30 mins each day, 3 days	EA improves motor impairment via inhibition of microglia-mediated neuroinflammation in the sensorimotor cortex after ischemic stroke	[26]
SAP	Sodium taurocholate-induced SAP rat model	ST25	MA or EA, 2–100 Hz and 2 mA, twice after SAP induction	Both MA and EA might have a therapeutic effect on rats with SAP through inhibition of NF-*κ*B expression and a reduction in the release of proinflammatory cytokines	[27]
Traumatic injury	Surgical trauma rat model	ST36, EX-LE7	EA, 2 Hz and 60 Hz alternating polarity for 30 mins, once after surgery	EA inhibits apoptosis of splenic lymphocytes in traumatized rats through modulation of the TNF-*α*/NF-*κ*B signaling pathway	[36]
	Feeney's free fall epidural impact method, TBI rat model	GV20, GV25, GV16, GV15, LI4	MA, 15 mins, thrice	Acupuncture has a bidirectional regulatory effect on the TLR2/4-NF-*κ*B signaling pathway-related genes TLR2, TLR4, and NF-*κ*B in the TBI rat cortex, promoting their expression in the early stage and inhibiting it in the later stage	[37]
VD	CMi rat model	ST36	Verum acupuncture	Acupuncture could protect cognitive function against oxidative stress induced by CMi, which is partially associated with suppression of NF-*κ*B-p53 activation	[38]

MA: manual acupuncture; EA: electroacupuncture; OVA: ovalbumin; DNCB: 1-chloro-2,4-dini-trobenzene; DNFB: 2,4-dinitrofluorobenzene; CAG: chronic atrophic gastritis; MNNG: N-methyl-N′-nitro-N-nitrosoguanidine; I/R: ischemia/reperfusion; COPD: chronic obstructive pulmonary disease; HIBD: hypoxic-ischemic brain damage; PTX: paclitaxel; OA: osteoarthritis; RA: rheumatoid arthritis; MACo: middle cerebral artery occlusion; SAP: severe acute pancreatitis; TBI: traumatic brain injury; VD: vascular dementia; CMi: cerebral multi-infarction.
